# Lateral swing flap of the nasal pyramid: A novel approach to the anterior skull base and review of surgical approaches

**DOI:** 10.1097/MD.0000000000046789

**Published:** 2025-12-26

**Authors:** Adel Azar, Ahmad Alkheder, Ahmad Mustafa

**Affiliations:** a Department of Otorhinolaryngology, Al-Mouwasat University Hospital, Damascus University, Damascus, Syria; b Faculty of Medicine, Damascus University, Damascus, Syria; c Faculty of Medicine, Syrian Private University, Damascus, Syria.

**Keywords:** lateral rhinotomy, novel approach, pediatric, skull base surgery, surgical approaches

## Abstract

**Rationale:**

Anterior skull base lesions in pediatric patients pose distinct surgical challenges owing to ongoing anatomical development and the necessity to preserve facial growth and cosmesis. Traditional approaches may involve significant morbidity or intracranial entry.

**Patient concerns:**

A 6-year-old boy presented with a several-month history of exophthalmos, lateral displacement of the right eye, and persistent nasal obstruction.

**Diagnoses:**

Non-contrast computed tomography and magnetic resonance imaging T2-weighted imaging revealed an extradural mass extending into the periorbital region and anterior skull base. Histopathological analysis identified the lesion as an aneurysmal bone cyst.

**Interventions:**

We employed a novel approach utilizing a vascularized nasal pyramid swing flap. The procedure involved en bloc mobilization of the nasal bones, cartilage, and overlying skin, facilitating wide multiaxial exposure of the anterior skull base without intracranial entry.

**Outcomes:**

Complete resection of the lesion was achieved without cerebrospinal fluid leak or intraoperative complications. Postoperatively, the patient experienced complete resolution of the symptoms with excellent cosmetic results.

**Lessons:**

Our approach demonstrates a tailored, minimally disruptive strategy for benign pediatric skull base lesions requiring extensive extradural access, balancing oncological efficacy with preservation of critical growth centers. Careful patient selection remains essential to avoid malignancies or intracranial involvement necessitating dural repair.

## 1. Introduction

Anterior skull base lesions in pediatric patients present unique surgical challenges due to the complex anatomy, proximity to critical neurovascular structures, and the imperative to minimize long-term functional and cosmetic morbidity.^[1]^ Traditional approaches, including lateral rhinotomy, craniofacial resection, and endoscopic endonasal techniques, aim to balance oncological efficacy with preservation of form and function.^[1–3]^ The lateral rhinotomy, initially described for sinonasal malignancies, provides direct access to the midface and anterior skull base but may involve significant soft tissue dissection and limited exposure for extensive lesions.^[1,3]^ In children, these approaches require additional considerations for craniofacial growth, psychosocial impact, and technical adaptations given the anatomical proportions.^[1]^ While endoscopic techniques have reduced invasiveness for midline lesions, laterally extending pathologies—particularly those involving the orbit, ethmoid roof, or frontal sinus—often necessitate open or combined approaches.^[2–4]^ Conventional osteotomies (e.g., medial/lateral) may still restrict visualization for extradural lesions with periorbital extension.^[1,2]^ When specifically considering the maxillary sinus, common pediatric approaches include the Caldwell–Luc procedure, endoscopic middle meatal antrostomy, and midfacial degloving. The Caldwell–Luc, while providing direct access, raises concerns regarding injury to unerupted dentition and facial growth centers.^[1,5]^ Endoscopic antrostomy is minimally invasive but offers limited working space and instrumentation angles in children, often insufficient for extensive lesion extirpation.^[2,3]^ The midfacial degloving approach, though avoiding facial scars, can compromise superior and posterior maxillary sinus access and carries a risk of septal complications affecting midfacial growth.^[1,3]^ These limitations are particularly pronounced when pathology extends beyond the sinus confines into the periorbital region and anterior skull base. Thus, innovations in access strategies that optimize exposure while preserving cosmesis are critical in pediatric skull base surgery.^[1,5]^

We describe a novel modification of the lateral rhinotomy using a nasal pyramid swing flap—comprising enbloc mobilization of nasal bones, cartilage, and skin—to access an expansive extradural aneurysmal bone cyst (ABC) in a young child. This technique addresses limitations of conventional approaches by providing wide, multiaxial exposure without compromising vascular integrity or facial growth potential, aligning with principles of minimally disruptive yet effective skull base surgery.

## 2. Case presentation

**Figure 1.**
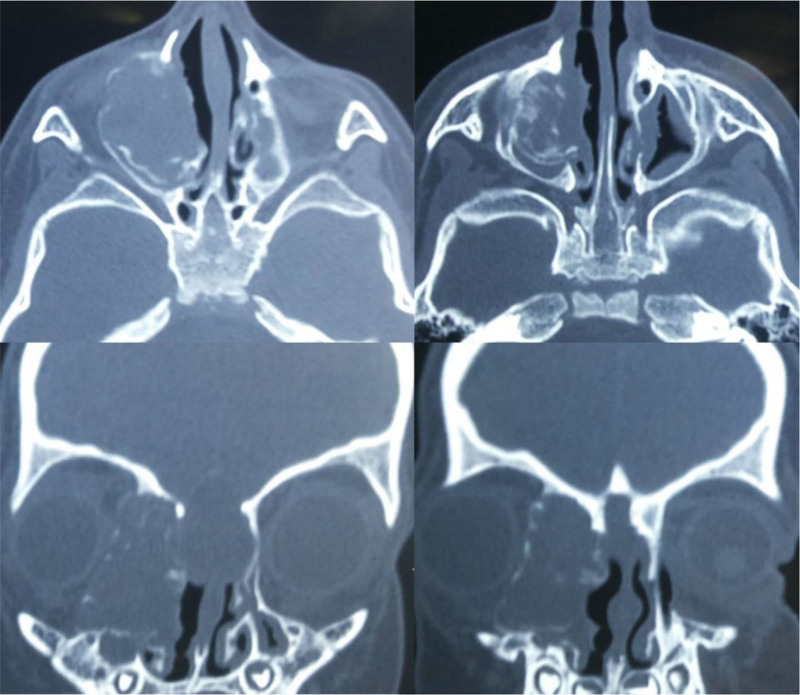
Non-contrast computed tomography revealed an extradural mass extending into the periorbital region and anterior skull base.

**Figure 2.**
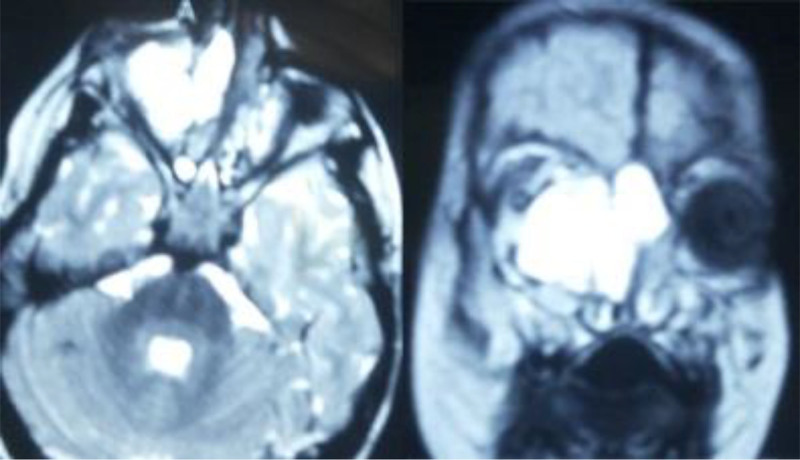
MRI T2-weighted imaging revealed a hyperintense lesion located in the periorbital area and the anterior skull base.

A 6-year-old boy presented with exophthalmos and lateral displacement of the right eye, accompanied by persistent nasal obstruction over several months. There were no neurological symptoms. Non-contrast computed tomography revealed an extradural mass extending into the periorbital region and anterior skull base (Fig. 1). MRI T2-weighted imaging demonstrated a bright lesion in the same area (Fig. 2).

**Figure 3.**
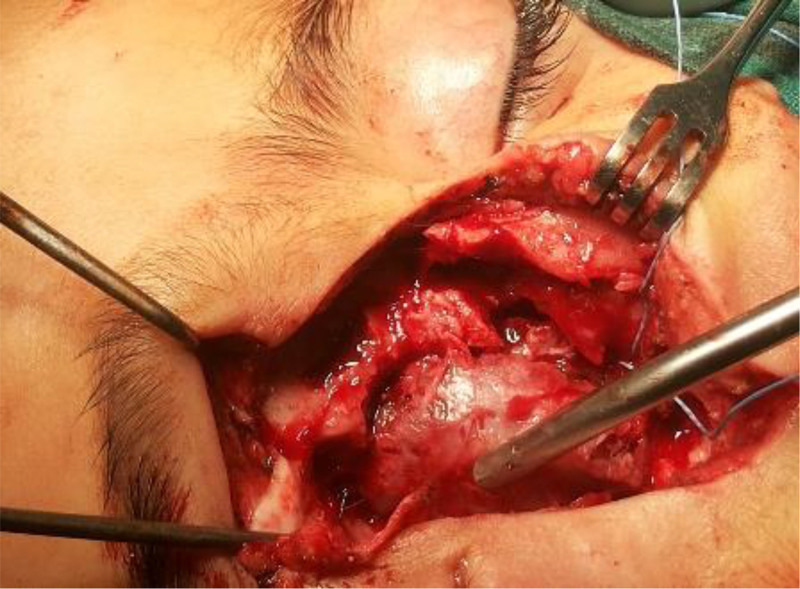
Complete exposure of the lateral component of the mass.

**Figure 4.**
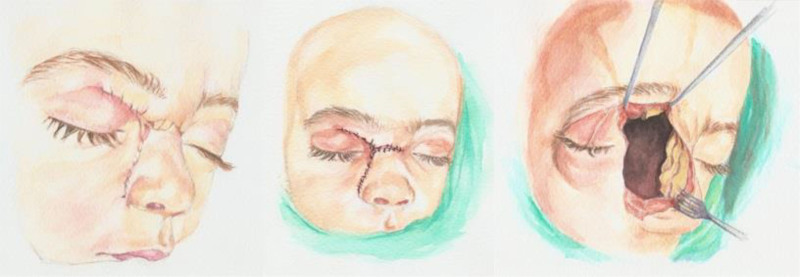
A schematic diagram illustrating the nasal pyramid swing flap.

Modified lateral rhinotomy approach: The procedure entailed making a right lateral rhinotomy incision, after which the right nasal bone and a segment of the frontal process of the maxilla were excised. The lateral component of the lesion was carefully dissected from the orbital periosteum due to a lamina papyracea defect. A frontonasal osteotomy was performed, along with a left lateral osteotomy. Additionally, septal cartilage was dissected from the nasal dorsum anterior to the lesion. This approach allowed creation of a nasal pyramid swing flap—comprising the nasal bones, cartilage, and overlying skin—that provided access to the anterior skull base. The flap was mobilized and rotated based on the left lateral mucosal and cutaneous tissue, with a basal connection to the columella, ensuring optimal visualization of the mass (Figs. 3 and 4). The lateral component of the lesion was excised first, followed by removal of the medial portion extending extradurally. The medial portion was carefully separated from the dura, where a local tear was identified. This was closed securely using figure-of-eight 0–5/0–4 Prolene sutures after achieving hemostasis. Surgicel was applied to address the defect in the cribriform plate of the ethmoid bone.

**Figure 5.**
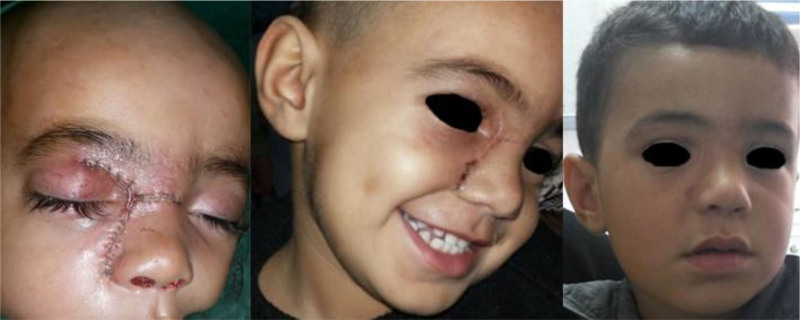
Cosmetic outcomes observed intraoperatively, at 2 weeks, and after 2 months, demonstrating excellent results.

Histopathological analysis identified the lesion as a aneurysmal bone cyst. Postoperatively, the patient experienced complete resolution of the symptoms with excellent cosmetic results (Fig. 5).

## 3. Discussion

Anterior skull base lesions in pediatric patients demand surgical approaches that balance maximal resection with minimal morbidity, given the unique vulnerabilities of developing anatomy and psychosocial considerations. The extradural aneurysmal bone cyst in this 6-year-old boy, extending into the periorbital region and anterior skull base, exemplifies the challenges of accessing laterally expansive pathologies while preserving critical structures. Traditional approaches each carry distinct advantages and limitations, as evidenced by the literature and our institutional experience.

Endoscopic endonasal approach (EEA): EEA offers minimally invasive access to midline anterior skull base lesions, avoiding external incisions and reducing brain retraction. It facilitates early devascularization of tumors and direct visualization of the cribriform plate, crista galli, and planum sphenoidale.^[2,4]^ However, its lateral reach is constrained by the midorbital line, optic nerves, and internal carotid arteries.^[4]^ In this case, the lesion’s periorbital and extradural lateral extension would have exceeded EEA’s working corridor, risking incomplete resection. Additionally, pediatric nasal dimensions limit instrument maneuverability, and large dural defects increase cerebrospinal fluid (CSF) leak risk (5%–20%) despite vascularized flaps.^[2,3,5]^

Midfacial degloving: This approach avoids facial scars by utilizing intraoral and intranasal incisions, providing broad exposure to the midface and sinonasal cavity.^[1,3]^ It is ideal for benign lesions without anterior cranial fossa invasion. However, its major limitation is compromised access to the superior septum and frontal sinus.^[1,3]^ In our patient, the lesion’s extradural skull base involvement and periorbital extension would have necessitated adjunctive osteotomies, increasing complexity. Furthermore, pediatric septal manipulation risks growth disturbance and saddle-nose deformity.^[1,5]^

Anterior fossa approach (bifrontal/subcranial): Bifrontal craniotomy enables wide exposure of the anterior skull base, facilitating enbloc resection of tumors with dural or intracranial involvement.^[1,2]^ It allows simultaneous intracranial and extracranial dissection and robust reconstruction using pericranial flaps.^[5]^ Drawbacks include frontal lobe retraction (risk of edema), anosmia, and frontal sinus complications (mucocele, CSF leak).^[1,5]^ For this extradural lesion, the approach would have been unnecessarily invasive, with higher risks of cosmetic deformity and developmental impact on the pediatric frontal bone.^[1,2]^

Extracranial transfrontal approach: This technique accesses the anterior fossa via osteotomies without intracranial dissection, minimizing brain manipulation.^[1,4]^ It suits lesions confined to the frontal sinus or orbital roof. However, exposure is restricted medially by the crista galli and cribriform plate.^[4]^ In our case, the lesion’s posterior ethmoidal and periorbital extension would have limited visibility, requiring endoscopic assistance. Pediatric applications are rare due to frontal sinus underdevelopment.^[1,4]^

Intracranial approaches (pterional/supraorbital): Supraorbital “keyhole” approaches provide direct corridors to the orbital roof and anterior clinoid with minimal soft tissue disruption.^[4]^ They are optimal for lateral suprasellar or orbital apex lesions but offer poor midline access.^[4]^ Drilling the cribriform plate or crista galli is anatomically unfeasible, and reconstruction options are limited.^[4]^ For this medial periorbital lesion, the approach would have necessitated significant brain retraction and failed to address septal/nasal involvement.^[4]^

Conventional lateral rhinotomy provides direct midface access but often requires soft tissue dissection and medial canthal detachment, risking telecanthus and scar contracture in children.^[1,3]^ Our modification—enbloc mobilization of the nasal pyramid (bones, cartilage, and skin) as a vascularized flap pedicled on the columella—overcame these limitations. By preserving the left lateral mucocutaneous supply, we maintained vascular integrity while achieving multiaxial exposure equivalent to midfacial degloving, without septal disruption.^[1,3]^ The technique enabled: Complete visualization of the extradural periorbital and ethmoidal components, circumventing EEA’s lateral constraints^[4]^; Avoidance of intracranial entry, reducing CSF leak risk versus transcranial routes^[5]^; Cosmetic preservation by eliminating facial incisions and minimizing growth center disruption.^[1,3]^ This approach aligns with pediatric principles of functional preservation and minimally disruptive access. The excellent cosmetic outcome underscores its utility for benign expansile lesions requiring wide but extradural exposure.

For anterior skull base defects post-aneurysmal bone cysts resection, vascularized reconstruction (e.g., nasoseptal or pericranial flaps) mitigates CSF leak risk.^[6]^ The “double-flap” technique—simultaneous pericranial and nasoseptal flaps—is optimal when adjuvant radiation is anticipated, enhancing durability in high-risk cases.^[6,7]^ Pediatric applications require meticulous preservation of growth centers to prevent midface hypoplasia.^[6,7]^

ABCs in the paranasal sinuses or skull base are exceptionally rare, accounting for < 3% of all ABCs, with only 89 cases documented in the largest systematic review to date.^[8]^ Their predilection for the ethmoid sinus (61%) and maxillary sinus (55%) underscores the diagnostic challenge, as symptoms like painless swelling (39%) and proptosis (38%) mimic more common pathologies.^[8]^ This rarity necessitates high clinical suspicion, particularly in pediatric patients with orbital displacement or nasal obstruction without neurological deficits.^[8]^ Orbital extension in ABCs correlates strongly with recurrence (hazard ratio 3.22, *P* = .03).^[8]^ Proptosis—a key presenting sign (38%)—was similarly linked to poor outcomes (hazard ratio 3.73, *P* = .01).^[8]^ This may reflect anatomical constraints hindering gross total resection or intrinsic biological aggression, though 67% of such cases still achieved complete resection.^[8]^ Vigilant surveillance is critical, as 93% of recurrences manifest within 24 months.^[8]^

Our philosophy of utilizing a tailored, tissue-preserving technique aligns with the evolving trend in pediatric skull base surgery towards minimizing morbidity without compromising efficacy. This principle is effectively demonstrated in the work of Gupta et al who reported successful management of juvenile nasopharyngeal angiofibroma using a combined transpalatal and endoscopic approach. Their technique, similar in spirit to our nasal pyramid swing flap, underscores the significant advantage of leveraging complementary surgical corridors to achieve complete tumor extirpation under direct visualization while preserving normal anatomy. This synergy between open and endoscopic principles provides a robust framework for managing select benign pediatric skull base pathologies.^[9]^

The successful execution of such procedures Is fundamentally predicated on meticulous preoperative planning and stringent intraoperative hemostasis. This is particularly paramount in the pediatric population, where physiological reserves are lower. The case detailed by Chmielik et al powerfully illustrates the critical importance of a comprehensive strategy—encompassing preoperative optimization, advanced hemodilution techniques, and precise surgical dissection—in managing a highly vascular skull base tumor in an adolescent who refused blood transfusion. Their experience reinforces that profound blood loss is not an inevitable consequence of resecting these lesions and validates the core tenet of our approach: achieving maximal exposure and definitive resection through anatomic planes with minimal blood loss, thereby circumventing the need for allogenic blood products.^[10]^

While successful here, this technique may be unsuitable for malignant tumors requiring enbloc resection or intracranial involvement necessitating dural repair. Combined approaches (e.g., EEA + supraorbital) could address such complexity,^[4]^ but carry higher morbidity. Furthermore, the approach relies on an intact and well-vascularized columellar pedicle, which could be compromised by prior surgery or trauma. In very young infants or patients with specific craniofacial syndromes, smaller nasal dimensions and less developed bone structure may pose technical challenges and limit the working corridor. Finally, the learning curve associated with the flap elevation and mobilization is not insignificant, requiring familiarity with advanced rhinoplasty techniques to ensure both viability and subsequent cosmetic reconstruction. Future innovations should refine flap design to enhance posterior fossa access and integrate intraoperative navigation for pediatric anatomic variations. The nasal pyramid swing flap modification of lateral rhinotomy offers a tailored, minimally disruptive approach for pediatric anterior skull base lesions with lateral periorbital extension. It balances wide exposure, vascular preservation, and cosmesis—addressing limitations of conventional techniques while aligning with the ethos of pediatric skull base surgery.

## 4. Conclusion

This novel modification of the lateral rhinotomy utilizing a vascularized nasal pyramid swing flap provided unparalleled multiaxial exposure for a complex pediatric extradural anterior skull base lesion, while preserving critical growth centers and vascular integrity. The technique circumvented the limitations of conventional approaches by eliminating facial incisions and intracranial entry, thereby minimizing morbidity and eliminating cerebrospinal fluid leak risk. Excellent functional and cosmetic outcomes were achieved, underscoring its utility as a tailored, minimally disruptive strategy for benign expansile pathologies requiring wide extradural access. Future applications warrant careful patient selection, particularly avoiding lesions with malignant features or intracranial involvement necessitating dural repair.

## Acknowledgments

The authors sincerely appreciate the valuable work of Mrs. Suha Salloum for creating the schematic diagram illustrating the technique.

## Author contributions

**Methodology:** Adel Azar, Ahmad Alkheder.

**Project administration:** Ahmad Mustafa.

**Software:** Adel Azar, Ahmad Alkheder.

**Supervision:** Ahmad Mustafa.

**Validation:** Adel Azar, Ahmad Alkheder.

**Visualization:** Adel Azar, Ahmad Alkheder, Ahmad Mustafa.

**Writing – original draft:** Adel Azar, Ahmad Alkheder, Ahmad Mustafa.

**Writing – review & editing:** Adel Azar, Ahmad Alkheder, Ahmad Mustafa.
